# Abnormal regional homogeneity and amplitude of low frequency fluctuation in chronic kidney patients with and without dialysis

**DOI:** 10.3389/fnins.2022.1064813

**Published:** 2022-11-22

**Authors:** Huan Yu, Chaoyang Zhang, Yan Cai, Ning Wu, Kai Duan, Wenwei Bo, Ying Liu, Zitong Xu

**Affiliations:** ^1^Department of Radiology, Liangxiang Hospital, Beijing, China; ^2^Department of Nephrology, General Hospital of the Chinese People’s Liberation Army, Beijing, China; ^3^Department of Nephrology, The Affiliated Hospital of Yangzhou University, Yangzhou, China; ^4^Yanjing Medical College, Capital Medical University, Beijing, China

**Keywords:** chronic kidney disease, regional homogeneity, amplitude of low frequency, dialysis, resting-state functional magnetic resonance imaging

## Abstract

**Purpose:**

The study characterizes regional homogeneity (ReHo) and amplitude of low frequency fluctuations (ALFF) in abnormal regions of brain in patients of chronic kidney disease (CKD).

**Materials and methods:**

A total of 64 patients of CKD were divided into 26 cases of non-dialysis-dependent chronic kidney disease (NDD-CKD), and 38 cases of dialysis-dependent chronic kidney disease (DD-CKD). A total of 43 healthy controls (normal control, NC) were also included. All subjects underwent resting-state functional magnetic resonance imaging (rs-fMRI). ALFF and ReHo data was processed for monitoring the differences in spontaneous brain activity between the three groups. ALFF and ReHo values of extracted differential brain regions were correlated to the clinical data and cognitive scores of CKD patients.

**Results:**

Non-dialysis-dependent group has increased ALFF levels in 13 brain regions while that of DD group in 28 brain regions as compared with NC group. ReHo values are altered in six brain regions of DD group. ALFF is correlated with urea nitrogen and ReHo with urea nitrogen and creatinine. DD group has altered ReHo in two brain regions compared with NDD group. The differences are located in basal ganglia, cerebellar, and hippocampus regions.

**Conclusion:**

Abnormal activity in basal ganglia, cerebellar, and hippocampal regions may be involved in the cognitive decline of CKD patients. This link can provide theoretical basis for understanding the cognitive decline.

## Introduction

Chronic kidney disease (CKD) systematically impairs renal function and glomerular filtration rate persists at <60 mL/min/1.72 m^2^ for more than 3 months. Patients suffer decline in attention, processing speed, functioning, alertness, verbal, and visual learning, along with weakened cognitive function such as theory of mind (Drew et al., 2019; Vondracek et al., 2021). The resting-state functional magnetic resonance imaging (rs-fMRI) non-invasively reflects the intrinsic activity of brain and assists in exploring the pathogenesis of neurological disorders (Li et al., 2020).

The amplitude of low frequency fluctuations (ALFF) detects the intensity of variations in blood oxygen level-dependent (BOLD) signal of fMRI. This is a stable indicator in fMRI sequence that responds to the low frequency electrical activity of neurons at rest. This measures brain activity without cognitive load to localize the neural activity in specific regions and physiological states of brain (Li et al., 2017; Zhu et al., 2021; Zhou et al., 2022). Local coherence regional homogeneity (ReHo) analysis reflects the synchronization of whole-brain voxels in localized regions of brain activity state (Yang et al., 2021). Its measurements are positively correlated with coherence and centrality of regional brain activity (Ji et al., 2020).

Amplitude of low frequency fluctuations method distinguishes neural activation patterns in healthy and diseased populations (Zhang et al., 2021). ALFF can be correlated with clinical and psychological phenomena in fMRI. Resting-state neuronal electrical activity can be assessed early in the disease, i.e., it can reveal localized abnormal brain activity in patients with CKD (Luo et al., 2016; Zhang et al., 2017; Li P. et al., 2021).

The ALFF and ReHo studies on cognitive impairment in CKD patients are not well documented. Abnormal ReHo and fractional amplitude of low frequency fluctuations (fALFF) in the brain of CKD patients under diverse treatment modalities assists in exploring the abnormalities of local brain functional activity. The obtained images elucidate the pathophysiological mechanisms of cognitive dysfunction in CKD. We provide a preliminary theoretical foundation that emphasizes the clinical risk factors to regulate the brain activity of CKD patients, which may be an early imaging symbol of CKD patients’ cognitive ability.

## Materials and methods

### Data acquisition

Sixty-four patients with CKD were selected who attended Liangxiang Hospital of Capital Medical University from January 2021 to December 2021. They were divided as whether they received dialysis treatment or not. NDD-CKD group had 26 patients with 17 males and 9 females of mean age 56.88 ± 11.70. DD group had 38 patients with 18 males and 20 females of mean age 58.06 ± 11.07. Inclusion criteria for CKD patients was as follows: (1) CKD patients meeting the NKF KDOQI diagnostic criteria; (2) age ≥18 years; (3) receiving maintenance hemodialysis and continuous ambulatory peritoneal dialysis for >3 months; and (4) no infection or other complication in last 3 months (Smith et al., 2013; Scialla et al., 2021). The exclusion criteria included: (1) Contraindication to MRI examination; (2) previous neurological and psychiatric diseases; and (3) tumor history. Inclusion criteria for healthy volunteers was as follows: (1) Age, gender, and education match with the patients; and (2) normal renal function. Exclusion criteria were the same as those for CKD group. Subjects signed an informed consent form and study was approved by the Ethics Committee of Liangxiang Teaching Hospital of Capital Medical University (no. 2016174).

### Data collection

Demographic data regarding patients’ age, gender, and education was collected. Serum creatinine, urea nitrogen, blood uric acid, and hemoglobin tests were conducted. eGFR was calculated using modified CG formula, eGFR = 186 × (SCr/88.4)–1.154 × age–0.203 × (0.742, female). eGFR of <15 mL/(min. 1.73 m^2^) was included in DD-CKD group and 15–60 mL/(min. 1.73 m^2^) in NDD-CKD group.

Resting-state fMRI data acquisition: MRI scans were made on Magnetom Skyra 3.0T MRI scanner (Siemens, Germany) using 32-channel phased-array magnetic head coil. Participants underwent fMRI scans with parameters: Single excitation gradient echo-echo planar imaging (SS-GRE-EPI) sequence with repetition time TR = 2,000 ms, echo time TE = 30 ms, flip angle FA = 90°, matrix = 64 × 64, field of view FOV = 224 mm × 224 mm, layer thickness = 4 mm, number of layers = 36, number of repetitions = 180, fat suppression on, and parallel imaging factor = 2. Subjects were asked to close eyes, lie down, relax, and remain awake and calm during functional MRI data acquisition.

### Data processing and analysis

#### Methods

Analyzed pipelines using configurable connectives (C-PAC),^1^ a python-based pipeline tool involving AFNI, ANTs, FSL, and custom python code (Zou et al., 2008; Tustison et al., 2014; Pruim et al., 2015; Hartig et al., 2021).

#### Structural processing

Structural processing involved following steps: (1) Images de-skewed; (2) images repositioned to right-to-left, posterior-to-anterior, inferior-to-upper (RPI); (3) cranial dissection performed; (4) cranially dissected brains normalized to Montreal Neurological Institute (MNI) 152 stereotactic space (1 mm isotropic) with linear and non-linear registration; (5) brain regions divided to gray matter, white matter, and cerebrospinal fluid; and (6) tissue segmentation of participants by priori knowledge of tissue in the standard space provided by FSL.

#### Functional processing

Functional preprocessing included following steps: (1) Removed first 10 time points; (2) performed slice timing correction; (3) images de-skewed; (4) images repositioned to right-to-left, back-to-front, down-to-up (RPI); (5) motion correction applied to mean image for obtaining motion parameters; (6) skull stripped; (7) global mean intensity normalized to 10,000; (8) white matter boundary-based linear transformation and FSL priori white matter tissue segmentation to register functional images to anatomical space; (9) removed motion artifacts using ICA-AROMA and partial component regression; (10) applied interference signal regression, including (a) mean of white matter signal (WM), (b) mean of cerebrospinal fluid signal (CSF), (c) 24 motion parameters (six head motion parameters, previous time point of 6 head motion parameters, and 12 corresponding squared terms), (d) linear trend of time series, and (e) global signal for one set of strategies; (11) bandpass time filtering from −0.01 Hz to 0.1 Hz; and (12) *z*-score followed by smoothing (FWHM = 6.0 mm).

#### Functional indicators

The fALFF in frequency of 0.01–0.1 Hz, and ReHo for 27 neighboring voxels, were generated.

### Statistical analysis

Two-samples *t*-test in DPABI^2^ software compared NDD-CKD vs. NC, DD-CKD vs. NC, and NDD-CKD vs. NC. ReHo and ALFF values of DD-CKD regressed off the effect of age and gender. Afterward, Gaussian Random Field (GRF) correction method was used for multiple comparison corrections. Voxel level significance of *P* < 0.001 was applied to generate clusters. Brain regions with cluster level significance of *P*-value < 0.05 were the regions of interest and their ALFF and ReHo values were extracted. SPSS 23.0 found out the relation of cognitive function with ALFF and ReHo using multiple linear regression models. *P*-value < 0.05 was considered a statistically significant difference.

## Results

### Comparison of demographic and clinical characteristics of dialysis-dependent, non-dialysis-dependent, and normal control groups

Total of 64 patients with CKD are included in this study, 26 in NDD, and 38 in DD. Baseline information of two groups is given in Tables 1, 2 the incidence of hypertension, diabetes mellitus and dyslipidemia in NDD-CKD group is higher, hemoglobin, red blood cells, protein, albumin, and phosphate are lower, and urea nitrogen and creatinine are higher than those in NC group (Table 1).

**TABLE 1:** Demographic of the CKD and NC groups.

	NC (*N* = 43)	CKD (*N* = 64)		T/X^2^ value			*P*-value		
		NDD-CKD group (*N* = 26)	DD-CKD group (*N* = 38)	A	B	C	A	B	C
**Demographics**									
Age (years)	55.64 ± 9.70	56.88 ± 11.70	58.06 ± 11.07	0.47	−0.99	−0.39	0.64	0.32	0.69
Male (%)	28 (66.67)	17 (65.38)	18 (58.06)	0.01	0.57	0.32	0.91	0.45	0.57
Duration of disease (years)	NA	3.00 ± 2.99	5.00 ± 4.50	NA	NA	−1.93	NA	NA	0.05
Hypertension (%)	5 (11.90)	23 (88.46)	28 (90.32)	38.86	44.28	0.05	0.00**	0.00**	0.82
Diabetes (%)	1 (2.38)	19 (73.08)	18 (58.06)	38.65	28.72	1.40	0.00**	0.00**	0.24
Dyslipidemia (%)	2 (4.76)	17 (65.38)	23 (74.19)	29.31	38.18	0.52	0.00**	0.00**	0.47

**p < 0.01.

A: Comparison between NC and NDD-CKD groups (NC vs. NDD-CKD).

B: Comparison between NC and DD-CKD groups (NC vs. DD-CKD).

C: Comparison between NDD-CKD group and DD-CKD group (NDD-CKD vs. DD-CKD).

**TABLE 2:** Clinical characteristics and cognitive scores of the CKD and NC groups.

	NC (*N* = 43)	CKD (*N* = 64)		T/X^2^ value			*P*-value		
		NDD-CKD group (*N* = 26)	DD-CKD group (*N* = 38)	A	B	C	A	B	C
**Laboratory tests**									
Hemoglobin, g/dl	134.95 ± 16.27	104.85 ± 23.66	112.61 ± 18.24	–5.71	5.51	–1.40	0.00**	0.00**	0.17
Erythrocyte pressure (%)	40.45 ± 4.24	31.85 ± 6.61	35.06 ± 5.78	–5.92	4.60	–1.95	0.00**	0.00**	0.06
Protein, g/dl	65.26 ± 5.50	59.96 ± 9.55	67.42 ± 7.33	–2.54	–1.44	–3.31	0.02*	0.16	0.002**
Albumin, g/dl	38.24 ± 2.72	32.50 ± 6.63	35.19 ± 4.62	–4.20	3.27	–1.75	0.00**	0.002**	0.09
Urea nitrogen, mg/dl	5.02 ± 1.36	14.30 ± 11.47	18.96 ± 5.02	4.11	–15.046	–1.920	0.000**	0.000**	0.064
Creatinine, mg/dl	67.26 ± 16.41	392.62 ± 328.65	969.35 ± 297.85	5.04	–16.844	–6.946	0.000**	0.000**	0.000**
Calcium, mg/dl	2.30 ± 0.11	2.19 ± 0.20	2.37 ± 0.19	–2.54	–1.71	–3.36	0.02*	0.09	0.001**
Phosphate, mg/dl	1.09 ± 0.27	1.45 ± 0.48	1.79 ± 0.73	3.97	–5.06	–2.01	0.00**	0.00**	0.049*
**Total MMSE score**	28.38 ± 1.27	27.00 ± 1.26	22.39 ± 2.75	–4.37	11.27	8.34	0.00**	0.00**	0.00**
**MoCA scale total score**	27.29 ± 1.60	25.46 ± 2.14	23.55 ± 2.28	–3.75	7.83	3.25	0.001**	0.00**	0.002**

*p < 0.05 and **p < 0.01.

### Comparison of amplitude of low frequency fluctuations between dialysis-dependent, non-dialysis-dependent, and normal control groups

**FIGURE 1:**
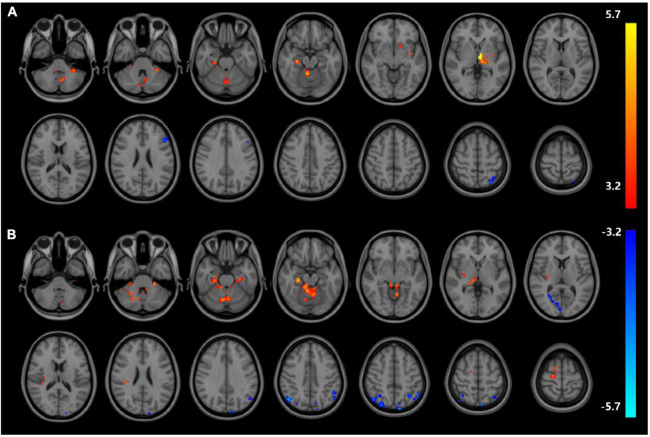
Results of ALFF **(A)** difference between NDD-CKD and NC groups; **(B)** difference between DD-CKD and NC groups; red indicates significantly increased ALFF and blue indicates significantly decreased ALFF. Corrected for GRF, voxel *P* < 0.001, cluster *P* < 0.05.

Analysis of patients corrected for age, gender, and education show that patients in NDD-CKD group compared to NC have more pronounced signal expression in left thalamus, left nucleus accumbens, left pallidum, left cerebellar regions 6 and 8, cerebellar earth regions 4, 5, 6, and 7, Frontal_Inf_TriL, right parahippocampal gyrus, right syrinx and left superior parietal gyrus (voxel *P* < 0.001, cluster *P* < 0.05). In DD-CKD group, bilateral cerebellar regions 3, 4, 5, 6, and right cerebellar region 8, left cerebellar region 9, right parahippocampal gyrus, right cerebellar horn, cerebellar earth regions 3, 4, 5, and 6, right thalamus, bilateral hippocampal gyrus, right sino-hypoglossal gyrus, Parietal_Inf_L, bilateral angular gyrus, bilateral superior occipital gyrus, left anterior temporal lobe, right superior parietal gyrus, and right insula are visible. ALFF values in cerebellar and thalamic regions are also increased in NDD-CKD and DD-CKD groups compared to NC. No significant differences are found in above regions of NDD-CKD compared to DD-CKD (Figure 1 and Tables 3, 4).

**TABLE 3:** Regions of significant differences in ALFF between NDD-CKD and NC.

Cluster	Number of voxel	Peak *T*-value	Peak MNI coordinates			Structure name
			x	y	z	
1	120	7.3	−6	−9	0	Thalamus_L, Putamen_L, Pallidum_L
2	98	5.0	−6	−63	−39	Cerebelum_8_L, Vermis_7, Vermis_6
3	45	–4.1	−48	24	24	Frontal_Inf_Tri_L
4	42	4.8	27	−24	−21	ParaHippocampal_R, Fusiform_R
5	37	–4.1	−30	−66	57	Parietal_Sup_L
6	36	6.0	9	−42	−48	Cerebelum_9_R
7	36	5.8	6	−48	−21	Vermis_4_5
8	34	4.5	−30	−42	−33	Cerebelum_6_L, Cerebelum_8_L

**TABLE 4:** Regions of significant differences in ALFF between DD-CKD and NC.

Cluster	Number of voxel	Peak *T*-value	Peak MNI coordinates			Structure name
			x	y	z	
1	513	5.6	9	−45	−21	Cerebelum_4_5_L, Cerebelum_4_5_R, Cerebelum_6_R, ParaHippocampal _R, Cerebelum_3_R, Cerebelum_Crus1_R, Vermis_6, Vermis_4_5, Vermis_3, Thalamus_R, Cerebelum_3_L, Hippocampus _R, Fusiform_R, Cerebelum_8_R, Cerebelum_6_L
2	89	–4.9	48	−66	42	Angular _R
3	88	–4.2	−54	−51	54	Parietal_Inf_L, Angular_L
4	64	5.0	−9	−42	−36	Cerebelum_9_L
5	53	–5.0	−9	−81	54	Occipital_Sup _L, Precuneus_L
6	52	4.4	21	−15	69	Precentral_R, Frontal_Sup_R
7	47	–4.2	27	−60	12	Calcarine _R
8	47	–4.3	21	−81	54	Parietal_Sup_R, Occipital_Sup_R
9	41	4.9	−24	−33	−33	Cerebelum_4_5_L, ParaHippocampal_L
10	39	4.5	36	−30	21	Insula _R

### Comparison of regional homogeneity in three groups

**FIGURE 2:**
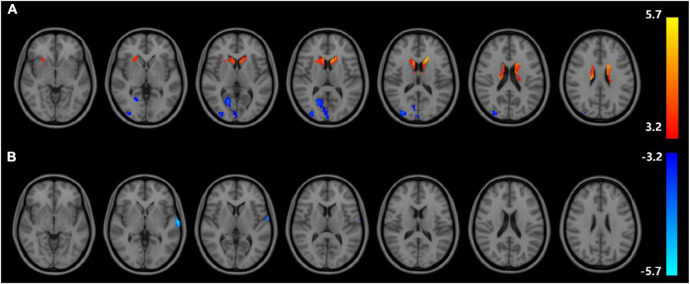
Regional homogeneity results; **(A)** difference between DD-CKD and NC groups, **(B)** difference between DD-CKD and NDD-CKD groups; red indicates a significant increase in ALFF, blue indicates a significant decrease in ALFF. Corrected for GRF, voxel *P* < 0.001, cluster *P* < 0.05.

Analyses corrected for age, gender, and education show that changes of ReHo expressions in six regions are statistically significant in DD-CKD compared to NC group (voxel *P* < 0.001, cluster *P* < 0.05), i.e., the ReHo expression is higher in bilateral caudate nucleus and right cisterna magna in DD-CKD than in NC group. ReHo of bilateral talar sulcus and right middle occipital gyrus is lower than that of NC group. ReHo is reduced between the left middle and left superior temporal gyrus in DD-CKD compared with NDD-CKD group (voxel *P* < 0.001, cluster *P* < 0.05). No significant differences are found between NDD-CKD and NC groups (Figure 2 and Tables 5, 6).

**TABLE 5:** Regions of significant differences in ReHo between DD-CKD and NC.

Cluster	Number of voxel	Peak *T*-value	Peak MNI coordinates			Structure name
			x	y	z	
1	142	4.9	18	−18	24	Caudate _R, Putamen_R
2	131	5.5	−15	18	18	Caudate_L
3	125	–4.2	21	−63	6	Calcarine_R, Calcarine_L
4	72	–4.2	33	−87	15	Occipital_Mid _R

**TABLE 6:** Regions of significant differences in ReHo between DD-CKD and NDD-CKD.

Cluster	Number of voxel	Peak *T*-value	Peak MNI coordinates			Structure name
			x	y	z	
1	38	−4.4	−66	−12	0	Temporal_Mid _COPYL, Temporal_Sup _COPYL

### Correlation of amplitude of low frequency fluctuations and regional homogeneity with urea nitrogen and creatinine in chronic kidney disease patients

**FIGURE 3:**
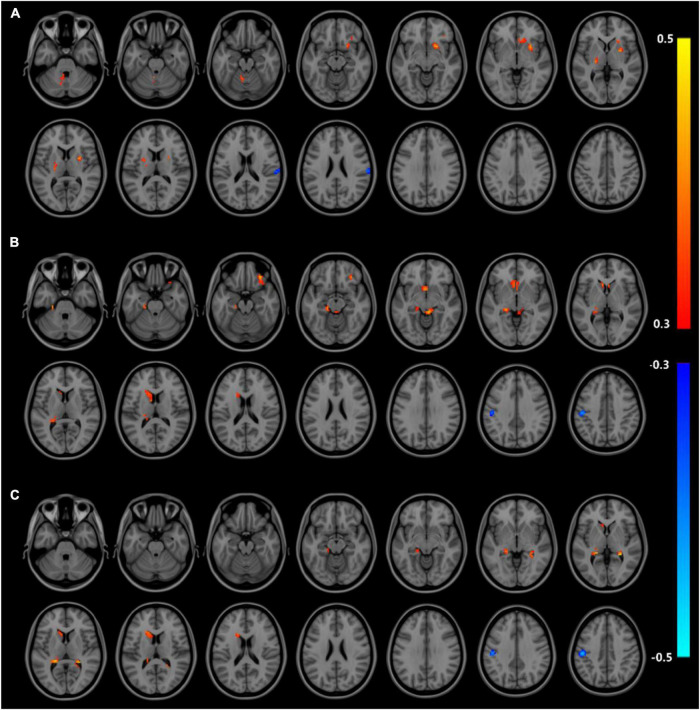
Results of correlation analysis of ALFF, ReHo with urea nitrogen and creatinine; **(A)** correlation of ALFF with urea nitrogen expression **(B)** correlation of ReHo with urea nitrogen expression **(C)** correlation of ReHo with creatinine expression; red represents positive correlation, blue represents negative correlation (corrected for GRF) voxel *P* < 0.001, cluster *P* < 0.05.

Correlation analysis of ALFF and ReHo with urea nitrogen and creatinine levels in subjects is performed with age and sex as covariates and values are corrected for GRF. Values are transformed to *z*-values before the analysis. The results reveal ALFF being correlated with urea nitrogen in nine regions: Bilateral nucleus accumbens, right caudate nucleus, left thalamus, left cerebellar regions 9, 4, 5, and 8, and right limbic gyrus with statistically significant differences (voxel *P* < 0.001, cluster *P* < 0.05). ReHo is correlated with urea nitrogen in the left caudate nucleus, bilateral olfactory bulb, left hippocampal gyrus, left parahippocampal gyrus, left cerebellar regions 4 and 5, right lingual gyrus, cerebellar earthworm region 3, left inferior parietal gyrus, left postcentral gyrus and Frontal_Inf_Orb_R with statistically significant differences. ReHo is associated with creatinine in the bilateral hippocampal gyrus, left parahippocampal gyrus, left caudate nucleus, left inferior parietal gyrus, and left postcentral gyrus (voxel P & lt; 0.001, cluster *P* < 0.05). No significant correlation is seen between ALFF and creatinine (Figure 3 and Tables 7–9).

**TABLE 7:** Correlation analysis of ALFF and urea nitrogen expression.

Cluster	Number of voxel	Peak *R*-value	Peak MNI coordinates			Structure name
			x	y	z	
1	123	0.45	24	12	−12	Putamen_R, Caudate_R
2	49	0.40	−21	−18	3	Thalamus_L, Putamen_L
3	41	0.39	−3	−51	−27	Cerebelum_9_L, Cerebelum_4_5_L
4	39	0.42	−30	−54	−54	Cerebelum_8_L
5	35	–0.41	63	−24	21	SupraMarginal_R

**TABLE 8:** Correlation analysis of ReHo and urea nitrogen expression.

Cluster	Number of voxel	Peak *R*-value	Peak MNI coordinates			Structure name
			x	y	z	
1	134	0.42	3	21	0	Caudate_L, Olfactory_L, Olfactory_R
2	90	0.45	−21	−24	−27	Hippocampus_L, ParaHippocampal_L, Cerebelum_4_5_L
3	47	0.44	6	−36	−9	Lingual_R, Vermis_3
4	43	–0.42	−48	−21	39	Parietal_Inf_L, Postcentral_L
5	39	0.47	63	−24	21	Frontal_Inf_Orb_R

**TABLE 9:** Correlation analysis of ReHo and creatinine expression.

Cluster	Number of voxel	Peak *R*-value	Peak MNI coordinates			Structure name
			x	y	z	
1	65	0.45	−18	−36	6	Hippocampus_L, ParaHippocampal_L
2	57	0.39	−15	18	15	Caudate_L
3	57	–0.45	−48	−21	39	Parietal_Inf_L, Postcentral_L
4	37	0.48	30	−39	3	Hippocampus_R

## Discussion

Cognition is the process through which individuals acquire and apply information, including attention, memory, verbal communication skills, executive skills, visual-spatial imagination, and orientation. Cognitive impairment refers to the damage of one or more of these abilities in varying degrees of severity (Drew et al., 2019; Viggiano et al., 2020). Its prevalence increases in CKD patients (Drew et al., 2017; Freire de Medeiros et al., 2020; Miglinas et al., 2020).

Spontaneous neural activity at rest can be analyzed by fMRI techniques (Li W. et al., 2021; Li et al., 2022). Herein, CKD patients are divided into dialysis and non-dialysis groups according to eGFR values. ALFF and ReHo are combined to study the changes in brain activity of CKD patients and correlating with clinical indicators. Increased ALFF values indicate enhanced neuronal activity and vice versa. The local coherence is a non-invasive method to find the activity of local functional neurons by studying the temporal coherence of BOLD signals of 27 spatially adjacent voxels in same time series. Abnormalities in synchronization or coordination indicate the impaired brain connectivity which is one of the early markers for diseases related to impaired brain neurological function. Results show that 13 brain regions in NDD and 28 in DD have increased ALFF values when compared to NC group. ReHo values are altered in six brain regions of DD group. DD group has altered ReHo in two brain regions compared with NDD group. The differences are in basal ganglia, cerebellum, and hippocampus regions. Basal ganglia relates to human cognitive, learning, motor, and memory functions. Neurons in basal ganglia are connected to cerebral cortex through associated circulation. Its lesions can damage this connection and cause cognitive dysfunction. New evidence suggests that cerebellum is involved in higher cognitive and emotional functions including visuospatial and emotional processing. Hippocampus is located between thalamus and medial temporal lobe. It is part of the limbic system and responsible for short-term memory storage, conversion, and orientation (Li et al., 2014; Fanouriakis et al., 2020). This suggests that abnormalities in brain functional activity may be influenced by clinical indicators and are the structural basis of cognitive impairment in patients.

A clinical study involving 90 patients with ESRD noted (Ruebner et al., 2016): Cognitive function correlates with glomerular filtration rate as part of the target organ damage in CKD. Present study also finds that altered ALFF values in above mentioned corresponding areas are correlated with urea nitrogen, and ReHo values with urea nitrogen and creatinine, suggesting that cognitive impairment in CKD patients is associated with specific structural brain alterations. The increased ALFF in hippocampal, basal ganglia and cerebellar regions in CKD group may be a compensatory mechanism of brain during cognitive decline. It is hypothesized that this compensatory state may be replaced by hypo-activated state as the disease progresses and adaptive limits of brain are exceeded. The brain regions with abnormal ReHo values in this study do not overlap with those with abnormal ALFF. This suggests that two analyses are based on different neurophysiological mechanisms. ALFF reflects the intensity and ReHo the coherence of neural activity, suggesting that brain tissue in CKD patients exhibits regional plastic response to implicit structural damage.

As a single-center cross-sectional study, this work has some limitations, i.e., the sample size needs expansion and the subgroups require refining for better correlations. Follow-up cohort studies can further correlate the changes in renal function, cognitive function and brain structure.

## Conclusion

The findings herein show that altered renal function in CKD patients compromises the cognitive function and brain structure. For the first time, brain-imaging-behavior relationship in patients with kidney disease is assessed. A preliminary theoretical basis is provided to emphasize that aggregation of clinical risk factors modulates brain activity in patients with CKD. This can be an early imaging marker of cognitive decline in CKD patients.

## Data availability statement

The original contributions presented in this study are included in the article/supplementary material, further inquiries can be directed to the corresponding author.

## Author contributions

HY and CZ designed the study and drafted the manuscript. KD, WB, and YL collected the MRI data. NW and YC analyzed and interpreted the results of the data. ZX and YC revised the manuscript. All authors approved the final manuscript.

## Footnotes

1
https://fcp-indi.github.io/

2
http://rfmri.org/dpabi

## References

[B1] Drew D. A. Weiner D. E. Sarnak M. J. (2019). Cognitive Impairment in CKD: Pathophysiology, Management, and Prevention. *Am J Kidney Dis* 74 782–790. 10.1053/j.ajkd.2019.05.017 31378643 PMC7038648

[B2] Drew D. A. Weiner D. E. Tighiouart H. Duncan S. Gupta A. Scott T. (2017). Cognitive Decline and Its Risk Factors in Prevalent Hemodialysis Patients. *Am J Kidney Dis* 69 780–787. 10.1053/j.ajkd.2016.11.015 28131531 PMC5441943

[B3] Fanouriakis A. Kostopoulou M. Cheema K. Anders H. J. Aringer M. Bajema I. (2020). 2019 Update of the Joint European League Against Rheumatism and European Renal Association-European Dialysis and Transplant Association (EULAR/ERA-EDTA) recommendations for the management of lupus nephritis. *Ann Rheum Dis* 79 713–723. 10.1136/annrheumdis-2020-216924 32220834

[B4] Freire de Medeiros C. M. M. Diógenes da Silva B. R. Costa B. G. Sartori V. F. Meneses G. C. Bezerra G. F. (2020). Cognitive impairment, endothelial biomarkers and mortality in maintenance haemodialysis patients: a prospective cohort study. *Nephrol Dial Transplant* 35 1779–1785. 10.1093/ndt/gfaa040 32379316

[B5] Hartig R. Glen D. Jung B. Logothetis N. K. Paxinos G. Garza-Villarreal E. A. (2021). The Subcortical Atlas of the Rhesus Macaque (SARM) for neuroimaging. *Neuroimage* 235 117996. 10.1016/j.neuroimage.2021.117996 33794360

[B6] Ji L. Meda S. A. Tamminga C. A. Clementz B. A. Keshavan M. S. Sweeney J. A. (2020). Characterizing functional regional homogeneity (ReHo) as a B-SNIP psychosis biomarker using traditional and machine learning approaches. *Schizophr Res* 215 430–438. 10.1016/j.schres.2019.07.015 31439419

[B7] Li C. Su H. H. Qiu Y. W. Lv X. F. Shen S. Zhan W. F. (2014). Regional homogeneity changes in hemodialysis patients with end stage renal disease: in vivo resting-state functional MRI study. *PLoS One* 9:e87114. 10.1371/journal.pone.0087114 24516545 PMC3916321

[B8] Li M. Gao Y. Gao F. Anderson A. W. Ding Z. Gore J. C. (2020). Functional engagement of white matter in resting-state brain networks. *Neuroimage* 220 117096. 10.1016/j.neuroimage.2020.117096 32599266 PMC7594260

[B9] Li P. Mu J. Ma X. Ding D. Ma S. Zhang H. (2021). Neurovascular coupling dysfunction in end-stage renal disease patients related to cognitive impairment. *J Cereb Blood Flow Metab* 41 2593–2606. 10.1177/0271678X211007960 33853410 PMC8504946

[B10] Li W. Wang Z. Zhang L. Qiao L. Shen D. (2017). Remodeling Pearson’s correlation for functional brain network estimation and autism spectrum disorder identification. *Frontiers in neuroinformatics* 11:55. 10.3389/fninf.2017.00055 28912708 PMC5583214

[B11] Li W. Xu X. Wang Z. Peng L. Wang P. Gao X. (2021). Multiple Connection Pattern Combination From Single-Mode Data for Mild Cognitive Impairment Identification. *Front Cell Dev Biol* 9:782727. 10.3389/fcell.2021.782727 34881247 PMC8645991

[B12] Li W. K. Chen Y. C. Xu X. W. Wang X. Gao X. (2022). Human-Guided Functional Connectivity Network Estimation for Chronic Tinnitus Identification: A Modularity View. *IEEE J Biomed Health Inform* 26 4849–4858. 10.1109/JBHI.2022.3190277 35830394

[B13] Luo S. Qi R. F. Wen J. Q. Zhong J. H. Kong X. Liang X. (2016). Abnormal Intrinsic Brain Activity Patterns in Patients with End-Stage Renal Disease Undergoing Peritoneal Dialysis: A Resting-State Functional MR Imaging Study. *Radiology* 278 181–189. 10.1148/radiol.2015141913 26053309

[B14] Miglinas M. Cesniene U. Janusaite M. M. Vinikovas A. (2020). Cerebrovascular Disease and Cognition in Chronic Kidney Disease Patients. *Front. Cardiovasc. Med.* 7:96. 10.3389/fcvm.2020.00096 32582768 PMC7283453

[B15] Pruim R. H. R. Mennes M. van Rooij D. Llera A. Buitelaar J. K. Beckmann C. F. (2015). ICA-AROMA: A robust ICA-based strategy for removing motion artifacts from fMRI data. *Neuroimage* 112 267–277. 10.1016/j.neuroimage.2015.02.064 25770991

[B16] Ruebner R. L. Laney N. Kim J. Y. Hartung E. A. Hooper S. R. Radcliffe J. (2016). Neurocognitive Dysfunction in Children, Adolescents, and Young Adults With CKD. *Am J Kidney Dis* 67 567–575. 10.1053/j.ajkd.2015.08.025 26476795

[B17] Scialla J. J. Kendrick J. Uribarri J. Kovesdy C. P. Gutiérrez O. M. Jimenez E. Y. (2021). State-of-the-Art Management of Hyperphosphatemia in Patients With CKD: An NKF-KDOQI Controversies Perspective. *Am J Kidney Dis* 77 132–141. 10.1053/j.ajkd.2020.05.025 32771650 PMC8109252

[B18] Smith S. M. Beckmann C. F. Andersson J. Auerbach E. J. Bijsterbosch J. Douaud G. (2013). Resting-state fMRI in the Human Connectome Project. *Neuroimage* 80 144–168. 10.1016/j.neuroimage.2013.05.039 23702415 PMC3720828

[B19] Tustison N. J. Cook P. A. Klein A. Song G. Das S. R. Duda J. T. (2014). Large-scale evaluation of ANTs and FreeSurfer cortical thickness measurements. *Neuroimage* 99 166–179. 10.1016/j.neuroimage.2014.05.044 24879923

[B20] Viggiano D. Wagner C. A. Martino G. Nedergaard M. Zoccali C. Unwin R. (2020). Mechanisms of cognitive dysfunction in CKD. *Nat Rev Nephrol* 16 452–469. 10.1038/s41581-020-0266-9 32235904

[B21] Vondracek S. F. Teitelbaum I. Kiser T. H. (2021). Principles of Kidney Pharmacotherapy for the Nephrologist: Core Curriculum 2021. *Am J Kidney Dis* 78 442–458. 10.1053/j.ajkd.2021.02.342 34275659

[B22] Yang F. Ma H. Yuan J. Wei Y. Xu L. Zhang Y. (2021). Correlation of abnormalities in resting state fMRI with executive functioning in chronic schizophrenia. *Psychiatry Res* 299 113862. 10.1016/j.psychres.2021.113862 33735738

[B23] Zhang X. D. Jiang X. L. Cheng Z. Zhou Y. Xu Q. Zhang Z. Q. (2017). Decreased Coupling Between Functional Connectivity Density and Amplitude of Low Frequency Fluctuation in Non-Neuropsychiatric Systemic Lupus Erythematosus: a Resting-Stage Functional MRI Study. *Mol Neurobiol* 54 5225–5235. 10.1007/s12035-016-0050-9 27578013

[B24] Zhang Z. Bo Q. Li F. Zhao L. Wang Y. Liu R. (2021). Increased ALFF and functional connectivity of the right striatum in bipolar disorder patients. *Prog Neuropsychopharmacol Biol Psychiatry* 111 110140. 10.1016/j.pnpbp.2020.110140 33068681

[B25] Zhou Y. Yu R. Ai M. Cao J. Li X. Hong S. (2022). A Resting State Functional Magnetic Resonance Imaging Study of Unmedicated Adolescents With Non-suicidal Self-Injury Behaviors: Evidence From the Amplitude of Low-Frequency Fluctuation and Regional Homogeneity Indicator. *Front Psychiatry* 13:925672. 10.3389/fpsyt.2022.925672 35782416 PMC9247173

[B26] Zhu J. Xu C. Zhang X. Qiao L. Wang X. Zhang X. (2021). Altered amplitude of low-frequency fluctuations and regional homogeneity in drug-resistant epilepsy patients with vagal nerve stimulators under different current intensity. *CNS Neurosci Ther* 27 320–329. 10.1111/cns.13449 32965801 PMC7871792

[B27] Zou Q. H. Zhu C. Z. Yang Y. Zuo X. N. Long X. Y. Cao Q. J. (2008). An improved approach to detection of amplitude of low-frequency fluctuation (ALFF) for resting-state fMRI: fractional ALFF. *J Neurosci Methods* 172 137–141 10.1016/j.jneumeth.2008.04.012 18501969 PMC3902859

